# The predictive value of perioperative circulating markers on surgical complications in patients undergoing robotic-assisted radical prostatectomy

**DOI:** 10.1186/s12957-023-03049-y

**Published:** 2023-06-12

**Authors:** Haohua Lu, Chenhao Yu, Muzhapaer Maimaiti, Gonghui Li

**Affiliations:** Department of Urology, Sir Run Run Shaw Hospital, School of Medicine, Zhejiang University, Hangzhou, 310016 Zhejiang China

**Keywords:** Prostatectomy, Robotic surgery, Multiport, Systemic inflammation, Surgical complication

## Abstract

**Background:**

The occurrence of postoperative complications was associated with poor outcomes for patients undergoing robotic-assisted radical prostatectomy. A prediction model with easily accessible indices could provide valuable information for surgeons. This study aims to identify novel predictive circulating biomarkers significantly associated with surgical complications.

**Methods:**

We consecutively assessed all multiport robotic-assisted radical prostatectomies performed between 2021 and 2022. The clinicopathological factors and perioperative levels of multiple circulating markers were retrospectively obtained from the included patients. The associations of these indices with Clavien-Dindo grade II or greater complications, and surgical site infection were assessed using univariable and multivariable logistic regression models. Further, the models were validated for the overall performance, discrimination, and calibration.

**Results:**

In total, 229 patients with prostate cancer were enrolled in this study. Prolonged operative time could independently predict surgical site infection (OR, 3.39; 95% CI, 1.09–10.54). Higher RBC (day 1-pre) implied lower risks of grade II or greater complications (OR, 0.24; 95% CI, 0.07–0.76) and surgical site infection (OR, 0.23; 95% CI, 0.07–0.78). Additionally, RBC (day 1-pre) independently predicted grade II or greater complications of obese patients (*P* value = 0.005) as well as those in higher NCCN risk groups (*P* value = 0.012). Regarding the inflammatory markers, NLR (day 1-pre) (OR, 3.56; 95% CI, 1.37–9.21) and CRP (day 1-pre) (OR, 4.16; 95% CI, 1.69–10.23) were significantly associated with the risk of grade II or greater complications, and both the indices were independent predictors in those with higher Gleason score, or in higher NCCN risk groups (*P* value < 0.05). The NLR (day 0-pre) could also predict the occurrence of surgical site infection (OR, 5.04; 95% CI, 1.07–23.74).

**Conclusions:**

The study successfully identified novel circulating markers to assess the risk of surgical complications. Postoperative increase of NLR and CRP were independent predictors for grade II or greater complications, especially in those with higher Gleason score, or in higher NCCN risk groups. Additionally, a marked decrease of RBC after the surgery also indicated a higher possibility of surgical complications, especially for the relatively difficult procedures.

**Supplementary Information:**

The online version contains supplementary material available at 10.1186/s12957-023-03049-y.

## Background

Prostate cancer is the second most common cancer among males around the world [1]. For men with organ-confined prostate cancer, radical prostatectomy (RP) is a principal curative treatment [2, 3]. In recent decades, robotic-assisted radical prostatectomy (RARP) has become a standard and widely used procedure for surgical management of prostate cancer [2–4]. Despite the development of da Vinci SP system for single-port surgery [5], RARP performed with multiport surgical system (MP-RARP) is still the most dominant approach [6].

Compared with retropubic radical prostatectomy (RRP), the minimally invasive procedures were reported to improve perioperative outcomes [4, 7, 8], but the robotic surgeries are still coupled with a complication rate ranging from 3 to 26% [9]. The occurrence of postoperative complications was associated with postoperative stay, cost of hospitalization, and disability [10, 11] and could even affect long-term outcomes [12]. Therefore, the development of an early prediction system for postoperative complications could alert clinicians in advance and reduce the potential risks of poor outcomes.

Currently, accumulating studies indicated that systemic inflammation played a critical role in the development and progression of cancer [13]. In line with this theory, multiple circulating biomarkers such as C-reactive protein (CRP) [14, 15], lymphocyte [16], systemic immune-inflammation index (SII) [17–19], neutrophil–lymphocyte ratio (NLR) [20, 21], platelet-lymphocyte ratio (PLR) [22, 23], lymphocyte-monocyte ratio (LMR) [22], and lymphocyte-CRP ratio (LCR) [24, 25] have been repeatedly reported to show powerful prognostic value. In addition to the survival outcomes, several studies have also identified strong associations between the systemic inflammatory biomarkers and postoperative complications [25–27]. The previous studies similarly leveraged preoperative systemic inflammatory status to predict the postoperative outcomes, but the preoperative status alone might overlook the impacts of surgery on postoperative outcomes. Therefore, we questioned whether incorporating both preoperative and postoperative systemic inflammatory status in the model could improve the prediction performance.

In the current study, we sought to utilize the perioperative levels of multiple systemic inflammatory biomarkers to predict the surgical complications of patients undergoing RARP.

## Methods

### Patient population

Patients who underwent MP-RARP using da Vinci Xi robotic system at Sir Run Run Shaw Hospital, School of Medicine, Zhejiang University, between January 2021 and January 2022 were consecutively enrolled in the study. All surgeries were performed by 7 surgeons who are experienced in RARP and beyond their learning curve. All patients were diagnosed with prostatic adenocarcinoma by ultrasound-guided prostate biopsy. The patients who received neoadjuvant therapy, lacked accurate postoperative pathological staging, or with severe cardiopulmonary diseases were excluded. The study was conducted with the approval of the Medical Ethical Committees of Sir Run Run Shaw Hospital (approval number: 2023–0022).

### Clinical data collection and follow-up investigation

Baseline preoperative demographics and clinical characteristics of the patients were retrospectively collected. Tumor histology was classified using the American Joint Committee on Cancer (AJCC) staging manual [28]. The preoperative blood tests of the patients were performed within 1 week prior to the surgery. Based on the previous studies [17–25], we identified several circulating markers (white blood cell, WBC; red blood cell, RBC; hemoglobin, HB; platelet, Plt; neutrophil, N; lymphocyte, L; monocyte, M; albumin; CRP; fibrinogen) from the blood tests, and with which, we further generated additional combination markers (neutrophil to lymphocyte ratio, NLR; platelet to lymphocyte ratio, PLR; lymphocyte to monocyte ratio, LMR; systemic immune-inflammation index, SII; lymphocyte to CRP ratio, LCR; CRP to albumin ratio, CAR). The NLR, PLR, LMR, SII, LCR, and CAR were calculated as follows: NLR = neutrophil/lymphocyte, PLR = platelet/lymphocyte, LMR = lymphocyte/monocyte, SII = platelet × neutrophil/lymphocyte, LCR = lymphocyte/CRP, and CAR = CRP/albumin. Likewise, the values of these circulating markers from laboratory blood tests at the day of the operation (postoperative day 0, POD 0) as well as the following day (postoperative day 1, POD 1) were also collected. We further calculated the differences of the circulating markers between different points (pretreatment, POD 0, and POD 1) in the perioperative period as follows:

$$\begin{array}{c}\mathrm{Marker\ }\left(\mathrm{day }\ 0-\mathrm{pre}\right)=\mathrm{ Marker\ }\left(\mathrm{POD }\ 0\right)-\mathrm{ Marker\ }(\mathrm{pretreatment})\\ \mathrm{Marker\ }\left(\mathrm{day\ }1-\mathrm{pre}\right)=\mathrm{ Marker }\left(\mathrm{POD }\ 1\right)-\mathrm{ Marker\ }(\mathrm{pretreatment})\\ \mathrm{Marker\ }\left(\mathrm{day\ }1-\mathrm{day}\ 0\right)=\mathrm{ Marker\ }\left(\mathrm{POD }\ 1\right)-\mathrm{ Marker\ }(\mathrm{POD }\ 0)\end{array}$$

The primary outcome was grade II or greater complications in the 6-month period after the surgery categorized using Clavien-Dindo classification system [29]. The secondary outcome was surgical site infection (SSI) defined according to the CDC guideline [30].

### Statistical analysis

Continuous parametric variables are presented as mean with standard deviation (SD). Continuous nonparametric variables are shown as median with interquartile range (IQR). Categorical variables are presented as frequency and percentage. The correlations between the circulating markers were assessed using Spearman correlation test. The effects of the variables on postoperative complications were estimated by univariable logistic regression. Significant variables were further included in a multivariable logistic regression model based on a stepwise forward selection approach to assess the independent predictive value. Odds ratios (ORs) and 95% confidence intervals (95% CIs) were used to evaluate the prediction values of perioperative circulating markers for surgical complications. The model performance was evaluated in terms of overall performance, discrimination, and calibration [31]. Overall performance was evaluated with Brier Score (BS). Discrimination was assessed using the area under the curve (AUC) from the receiver operating characteristic (ROC) curves analysis and with a fivefold cross validation. Calibration was measured with the Hosmer–Lemeshow test. All analyses were performed using SPSS Statistics 25.0 software (IBM SPSS Inc., USA) and R statistical software version 4.1.1 (The R Foundation for Statistical Computing). A *P* value < 0.05 was considered as statistically significant.

## Results

### Patient characteristics

A total of 229 patients were enrolled in this study after applying the inclusion and exclusion criteria. Characteristics of the population are presented in Table 1. Of these participants, the average age was 68.2 ± 6.5 years and 41.5% were older than 70 years. The mean body mass index (BMI) was 23.9 ± 2.8 kg/m^2^. The median preoperative PSA was 10.6 (IQR: 7.8–17.6) ng/ml. Among the patients, 37 (16.2%) had previous abdominal surgery and 16 (7.0%) had hernia surgery. The pelvic lymph node dissection (PLND) was performed on 15 patients. Overall, 44 (19.2%) patients developed a surgical complication in the follow-up period, of which, 37 were grade ≥ II.

**Table 1:** Demographics and clinical characteristics of the included prostate cancer patients (*n* = 229)

Characteristics	Number (%)
Age (years)	68.2 ± 6.5
BMI (kg/m^2^)	23.9 ± 2.8
Preoperative PSA (ng/ml) (IQR)	10.6 (7.8, 17.6)
Smoking history (%)	
None	144 (62.9%)
Former	42 (18.3%)
Current	43 (18.8%)
Prostate size (cm^3^) (IQR)	39.9 (27.4, 45.1)
Prior/Previous surgeries	
abdominal surgery	37 (16.2%)
pelvic surgery	35 (15.3%)
hernia surgery	16 (7.0%)
Biopsy Gleason score	
6	72 (32.9%)
7	83 (37.9%)
≥ 8	64 (29.2%)
Clinical stage	
cT1	41 (21.9%)
cT2	129 (69.0%)
cT3	16 (8.6%)
cT4	1 (0.5%)^a^
NCCN risk group	
Low risk	31 (16.8%)
Favorable intermediate	35 (19.0%)
Unfavorable intermediate	41 (22.3%)
High/very high risk	77 (41.8%)
Charlson comorbidity index	
< 2	220 (96.1%)
≥ 2	9 (3.9%)
ASA score	
≤ 2	209 (91.3%)
> 2	20 (8.7%)

^a^For the case of clinical stage cT4, the postoperative pathological stage was pT3b

### Correlations between the perioperative circulating markers

The correlations between the perioperative circulating markers are shown in Fig. S1. As illustrated in Fig. S1, the perioperative fluctuation of WBC and N was significantly correlated (Spearman correlation > 0.95, *P* value < 0.001). In addition, *N* (day 0-pre) was strongly correlated with NLR (day 0-pre) (Spearman correlation = 0.79, *P* value < 0.001) and SII (day 0-pre) (Spearman correlation = 0.81, *P* value < 0.001), and among which, NLR (day 0-pre) and SII (day 0-pre) were also highly correlated (Spearman correlation = 0.95, *P* value < 0.001). When comparing the circulating markers at POD 1 and baseline, correlations were observed between HB (day 1-pre) and RBC (day 1-pre) (Spearman correlation = 0.97, *P* value < 0.001). Moreover, CRP (day 1-day 0) and CRP (day 1-pre) were strongly correlated (Spearman correlation = 0.99, *P* value < 0.001) as well.

### Associations of perioperative circulating markers with grade II or greater complications

In the univariable analysis, several clinicopathological factors were significantly associated with the risk of grade II or greater complications, including operative time ≥ 2 h (OR, 3.11; 95% CI, 1.39–6.94) and estimated blood loss > 50 ml (OR, 2.43; 95% CI, 1.13–5.24) (Table S1). In terms of circulating markers, significant associations between the complications and multiple factors were identified (Table S2- 4), such as NLR (day 0-pre) ≥ 11.5 (OR, 5.65; 95% CI, 1.71–18.64), NLR (day 1-pre) ≥ 5.2 (OR, 2.23; 95% CI, 1.01–4.92), SII (day 0-pre) ≥ 2100 (OR, 3.88; 95% CI, 1.16–13.00), LCR (day 0-pre) ≥ 1.8 (OR, 3.02; 95% CI, 1.11–8.18), RBC (day 1-pre) ≥  − 1 (OR, 0.25; 95% CI, 0.09–0.66), HB (day 1-pre) ≥  − 30 (OR, 0.33; 95% CI, 0.13–0.82), N (day 0-pre) ≥ 2.5 (OR, 2.82; 95% CI, 1.29–6.17), N (day 1-day 0) ≥  − 1.2 (OR, 0.40; 95% CI, 0.17–0.95), CRP (day 1-pre) ≥ 28 (OR, 2.69; 95% CI, 1.26–5.74), and CRP (day 1-day 0) ≥ 29 (OR, 2.97; 95% CI, 1.38–6.38).

**Fig. 1:**
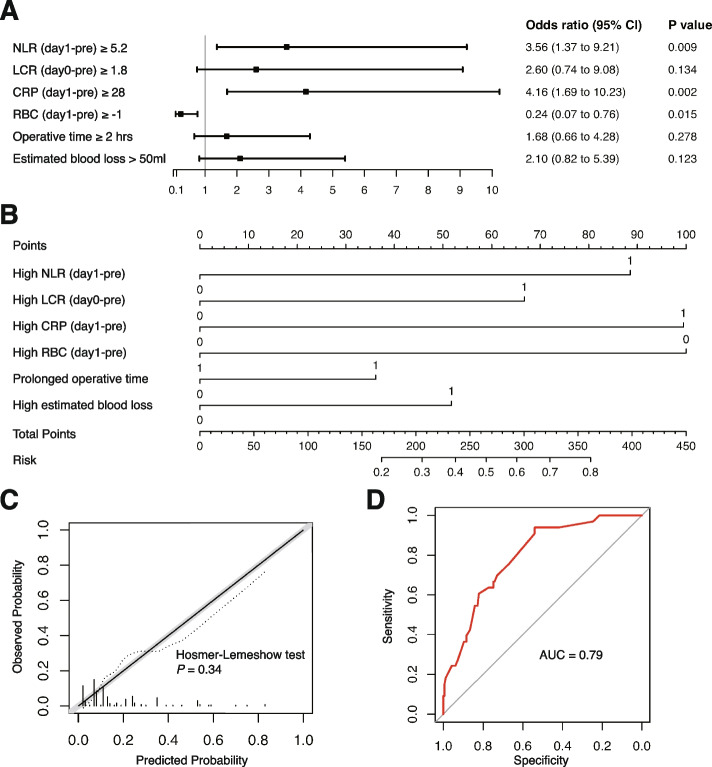
The multivariable model for grade II or greater complications after RARP. **A** Forest plot of the model for grade II or greater complication. **B** Nomogram with NLR (day 1-pre), LCR (day 0-pre), CRP (day 1-pre), RBC (day 1-pre), operative time, and estimated blood loss incorporated. **C** Calibration plot shows the relationship between predicted (solid line) and observed (dashed line) risk of grade II or greater complications. **D** ROC curve of the model for grade II or greater complication prediction

Given the Spearman coefficients between different circulating markers, we applied a threshold of 0.75 for |R| and excluded NLR (day 0-pre), SII (day 0-pre), N (day 0-pre), HB (day 1-pre), and CRP (day 1-day 0) to avoid a high correlation between the predictors. We incorporated NLR (day 1-pre), LCR (day 0-pre), CRP (day 1-pre), RBC (day 1-pre), operative time, and estimated blood loss in the multivariable model leveraging forward selection approach. High NLR (day 1-pre) and CRP (day 1-pre) as well as low RBC (day 1-pre) were independently associated with an increased risk of grade II or greater complications (Fig. 1A and B). The Brier Score of this model was 0.12. As presented in the calibration plot, the predicted risk calibrated well with observed outcomes (Hosmer–Lemeshow goodness-of-fit test *P* value = 0.34; Fig. 1C). Based on the ROC curve analysis, the AUC was 0.79 (Fig. 1D). In the cross validation, we obtained an average value of AUC of 0.76 (95% CI, 0.67–0.84).

**Fig. 2:**
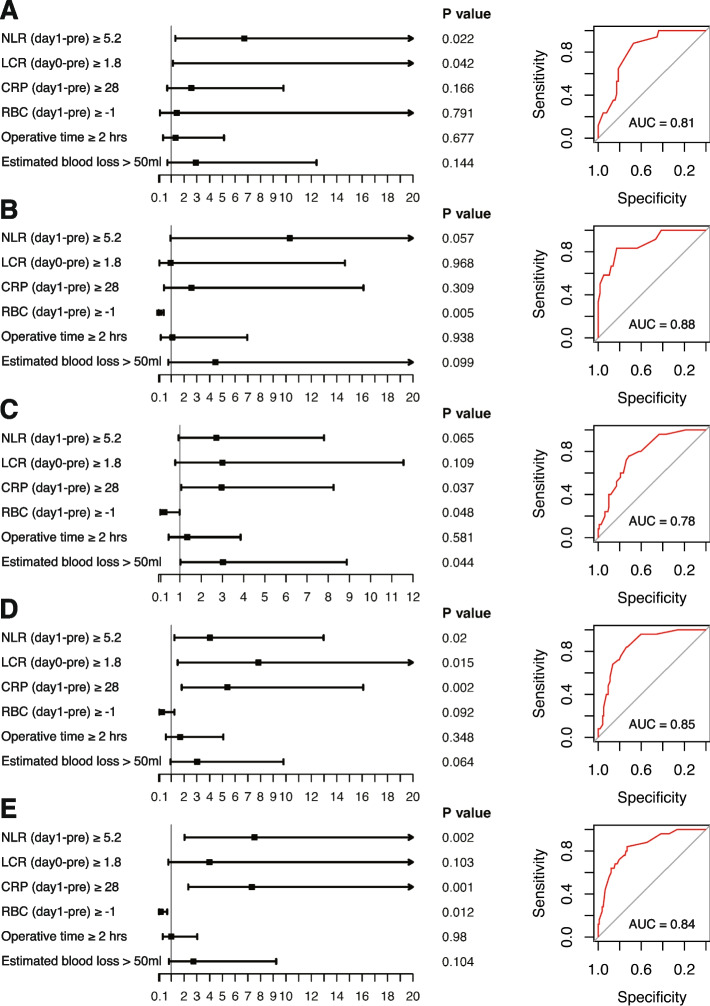
Multivariable models for grade II or greater complications in different subgroups. Multivariable analysis on grade II or greater complications was conducted in patients ≥ 70 years old (**A**), patients with BMI ≥ 25 kg/m^2^ (**B**), patients with prostate volume > 25 cm^3^ (**C**), patients with 7 or greater Gleason score (**D**), and patients in intermediate or high NCCN risk group (**E**). The forest plots and ROC curves of the models are shown

Further, we tested the model in several subgroups such as patients ≥ 70 years old, patients with BMI ≥ 25 kg/m^2^, patients with prostate volume > 25 cm^3^, patients with Gleason score 7 or greater and patients in moderate or high NCCN risk group (Fig. 2). High NLR (day 1-pre) was independently associated with higher risk of grade II or greater complications in older ones (*P* value = 0.022; Fig. 2A). And high NLR (day 1-pre) and CRP (day 1-pre) were both significantly associated with an increased risk of the complications in those with higher Gleason score (Fig. 2D) or in higher NCCN risk groups (Fig. 2E). Low RBC (day 1-pre) was an independent predictor for the complications in obese patients (*P* value = 0.005; Fig. 2B) as well as the patients in higher NCCN risk groups (*P* value = 0.012; Fig. 2E). For the patients with larger prostate, low RBC (day 1-pre) (*P* value = 0.048) and more estimated blood loss (*P* value = 0.044) could both independently predict the risk of grade II or greater complications (Fig. 2C).

### Associations of perioperative circulating markers with SSI

According to the univariable analysis, pathological stage T3 or T4 (OR, 2.31; 95% CI, 1.00–5.29) and operative time ≥ 2 h (OR, 4.55; 95% CI, 1.67–12.45) (Table S1) were significantly associated with SSI. Among the circulating markers, NLR (day 0-pre) ≥ 11.5 (OR, 3.79; 95% CI, 1.06–13.55), RBC (day 1-pre) ≥  − 1 (OR, 0.22; 95% CI, 0.08–0.61), HB (day 1-pre) ≥  − 30 (OR, 0.28; 95% CI, 0.11–0.71), N (day 0-pre) ≥ 2.5 (OR, 4.85; 95% CI, 1.77–13.29), M (day 0-pre) ≥ 0.07 (OR, 3.60; 95% CI, 1.57–8.26), and CRP (day 1-day 0) ≥ 29 (OR, 2.37; 95% CI, 1.01–5.55) were identified as significant predictors for SSI (Table S2- 4).

**Fig. 3:**
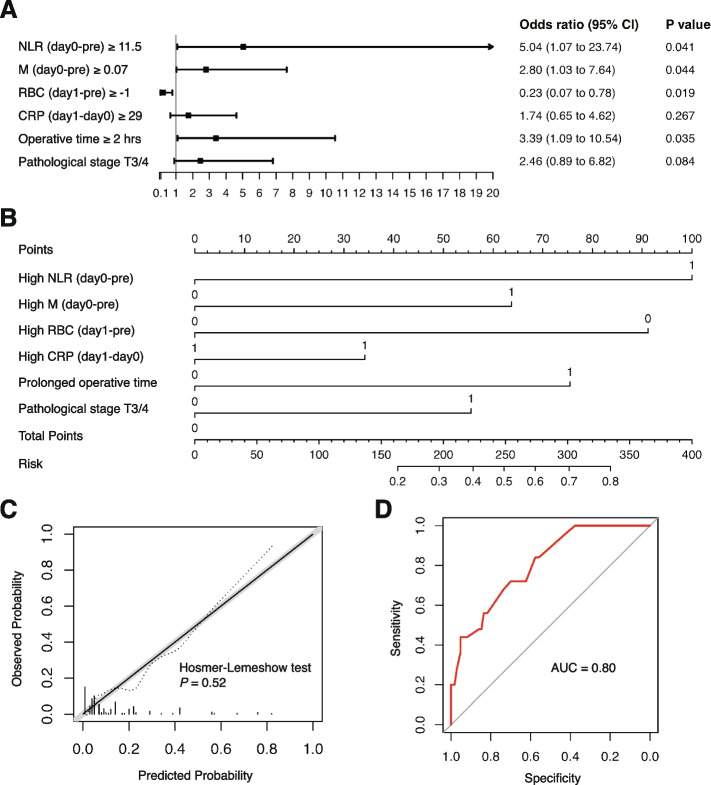
The multivariable model for SSI after RARP. **A** Forest plot of the SSI risk prediction model. **B** Nomogram with NLR (day 0-pre), M (day 0-pre), RBC (day 1-pre), CRP (day 1-day 0), operative time, and pathological stage incorporated. **C** Calibration plot shows the relationship between predicted (solid line) and observed (dashed line) risk of SSI. **D** The ROC curve of the SSI risk prediction model

Based on the Spearman correlation test result, we excluded N (day 0-pre) and HB (day 1-pre) with a threshold of 0.75 for |R|. Through a forward selection approach, we finally included NLR (day 0-pre), M (day 0-pre), RBC (day 1-pre), CRP (day 1-day 0), operative time, and pathological stage T3/4 in the multivariable logistic model. High NLR (day 0-pre) (OR, 5.04; 95% CI, 1.07–23.74) and M (day 0-pre) (OR, 2.80; 95% CI, 1.03–7.64), and long operative time (OR, 3.39; 95% CI, 1.09–10.54) independently predicted increased likelihood of SSI, while high RBC (day 1-pre) (OR, 0.23; 95% CI, 0.07–0.78) was an independent predictor for a decreased risk of this outcome (Fig. 3A and B). The Brier Score of this model was 0.089. The predicted and observed risks of SSI are illustrated as a calibration plot, and the Hosmer–Lemeshow goodness-of-fit test resulted in a *P* value of 0.52. The AUC of the ROC curve was 0.80 (Fig. 3D), and the mean AUC obtained from the cross-validation was 0.75 (95% CI, 0.61–0.90).

## Discussion

In the present study, we aimed to identify novel and simple indices to help surgeons evaluate patients for the risk of surgical complications. We analyzed the associations between circulating markers and the complications on patients undergoing RARP and further examined the relationships using the method of cross-validation.

Among the surgical factors, prolonged operative time (≥ 2 h) were independent predictors for SSI. In contrast, the characteristics of patients and cancer such as age, BMI, surgical history, and T stage might play a relatively minor role in surgical complications. Prolonged operative time has been repeatedly validated to be associated with poor outcomes following surgeries, including surgical complications [32, 33]. Integrating the evidence and the results in our study (Fig. 3A), operative time seemed to be a critical factor for the infectious outcomes of surgical patients.

As an indicator of blood loss in the perioperative period, RBC (day 1-pre) played an important role in predicting grade II or greater complications as well as SSI. And it also seemed to be a good predictor for postoperative complications in obese patients, and those with large prostate or in higher NCCN risk groups (Fig. 2B, C, and E). Higher values of RBC (day 1-pre) implied a reduced possibility of perioperative anemia, and therefore, a lower risk of a series of poor outcomes [34]. Consequently, it might be meaningful to proactively monitor the patients for decreasing RBC in the perioperative period and provide proper medical intervention. As demonstrated in a previous research, BMI, prostate volume, preoperative PSA level, middle lobe protrusion, and clinical stage were integrated to construct a scoring system which could successfully predict surgical difficulty [35]. Therefore, combining the results shown in Fig. 2B, C, and E, we could speculate that RBC (day 1-pre) might be a valuable predictor in the prostatectomies which are relatively difficult.

In terms of the systemic inflammatory markers, we identified several markers as critical predictors for surgical complications in peripheral blood, among which, NLR and CRP played a pivotal role. As demonstrated in Figs. 1 and 2, the elevated NLR and CRP during the perioperative period predicted an increased risk of grade II or greater complications especially in those with higher Gleason score or in higher NCCN risk groups. For the SSI, a marked increase of NLR in POD 0 compared with pretreatment was also an independent risk factor (Fig. 3A).

A series of studies have confirmed the predictive value of NLR for postoperative outcomes. An elevated NLR was associated with poor prognosis after surgery [20, 21, 36–42] as well as higher risk of postoperative complications [43, 44], which was also validated in meta-analyses [45–47]. Traditionally, it is believed that inflammation is the stress response to cellular or tissue injury [48]. Therefore, as an injury to tissue, surgery is undoubtedly coupled with the inflammatory response and could elevate the level of the inflammatory markers. The neutrophil is the pivotal initiator of tissue destruction cascades [49], while the lymphocyte is critical to the host cell-mediated cytotoxic immunity [50]. Incorporating the counts of neutrophil and lymphocyte, the elevation of NLR indicated a high level of systemic inflammation and stress [51–54], which might relate with a perturbed immune homeostasis [53], and could be an alert of poor clinical outcomes of the patients.

Increasingly synthesized in the systemic inflammation, CRP is regarded as a representative acute-phase reactant reflecting the inflammatory process [25, 55–57]. Accumulating studies have investigated the values of CRP in risk assessment. It was reported that preoperative as well as postoperative serum CRP are both correlated with postoperative outcomes of patients with cancer [14, 15, 37, 58–60]. In our study, CRP was strongly associated with grade II or greater complications, while for SSI, NLR seemed to be a better indicator compared with CRP.

The current study has several limitations. Firstly, given the relatively small sample size, our models were not externally validated. Secondly, the survival outcomes were not available in the clinical data due to the limited follow-up time. Therefore, it demands further investigations on the associations between the circulating markers and prognosis of these patients. Additionally, in view of the recent advance in single-port surgeries, a future study concerning single-port RARP (SP-RARP) would be valuable.

## Conclusions

The study successfully established models which used novel circulating markers to assess the risk of surgical complications. A postoperative elevation of NLR and CRP could predict an increased risk of grade II or greater complications, especially in those with higher Gleason score, or in higher NCCN risk groups. In terms of SSI, NLR seemed to be a better indicator than CRP. In addition, a decrease of RBC during perioperative period also indicated a higher possibility of surgical complications, especially for the relatively difficult procedures of RARP. The values of the circulating markers which could be easily accessed during hospitalization might be novel indices for the surgeons and guide the inpatient care and treatments.

## Supplementary Information

**Additional file 1: Figure S1.** Spearman correlation between the perioperative levels of circulating markers.

**Additional file 2: Table S1.** Univariable analysis of the associations between clinicopathological factors and the primary and secondary outcomes. OR: odds ratio; CI: confidence interval. **Table S2-4.** Univariable analysis of the associations between circulating markers and the primary and secondary outcomes. OR: odds ratio; CI: confidence interval.

## Data Availability

The datasets used and/or analyzed during the current study are available from the corresponding author on reasonable request.
