# Supplementary figures and images for: The predictive value of perioperative circulating markers on surgical complications in patients undergoing robotic-assisted radical prostatectomy
Source: World J Surg Oncol. 2023 Jun 12;21:179. doi: 10.1186/s12957-023-03049-y (PMC10258943; doi:10.1186/s12957-023-03049-y)

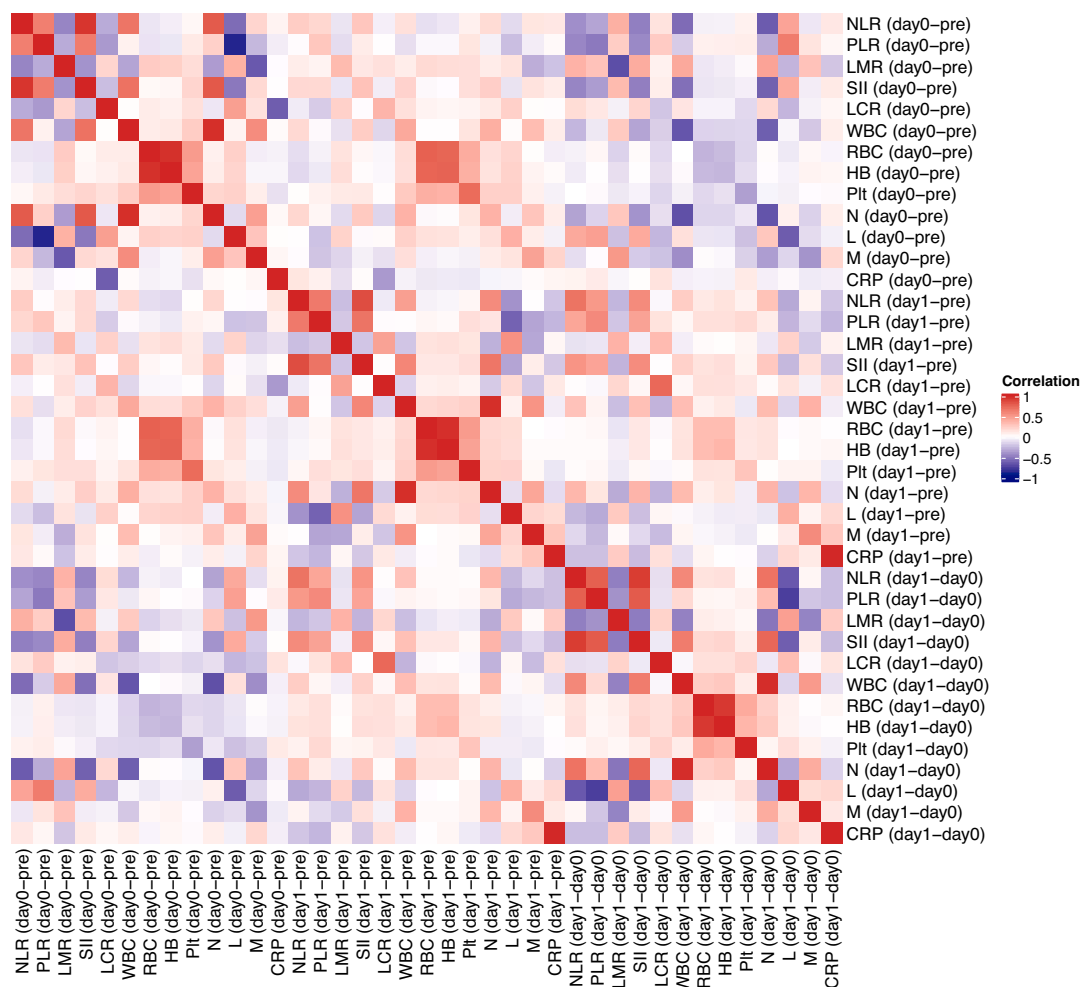

**Figure S1. Spearman correlation between the perioperative levels of circulating markers.**

Supplement: Supplementary file 1 — Additional file 1: Figure S1. Spearman correlation between the perioperative levels of circulating markers. [file 12957_2023_3049_MOESM1_ESM.pdf]
