# Supplementary material for: The predictive value of perioperative circulating markers on surgical complications in patients undergoing robotic-assisted radical prostatectomy
Source: World J Surg Oncol. 2023 Jun 12;21:179. doi: 10.1186/s12957-023-03049-y (PMC10258943; doi:10.1186/s12957-023-03049-y)
Supplement: Supplementary file 2 — Additional file 2: Table S1. Univariable analysis of the associations between clinicopathological factors and the primary and secondary outcomes. OR: odds ratio; CI: confidence interval. Table S2-4. Univariable analysis of the associations between circulating markers and the primary and secondary outcomes. OR: odds ratio; CI: confidence interval. [file 12957_2023_3049_MOESM2_ESM.pdf]

Table S1. Univariable analysis of the associations between clinicopathological factors and the primary and secondary outcomes. OR: odds ratio; CI: confidence interval.

		Grade II or greater complication		Surgical site infection	
		**OR (95% CI)**	**P**	**OR (95% CI)**	**P**
**Patient characteristics**					
Age	<70	reference		reference	
	≥70	1.61 (0.80-3.27)	0.19	1.26 (0.57-2.78)	0.57
Smoke	no	reference		reference	
	yes/ex	1.04 (0.50-2.14)	0.92	0.78 (0.34-1.81)	0.56
BMI	<25	reference		reference	
	≥25	1.03 (0.49-2.15)	0.95	1.26 (0.56-2.83)	0.58
Charlson comorbidity index	0	reference		reference	
	1	0.62 (0.24-1.58)	0.31	0.73 (0.26-2.03)	0.54
	≥2	0.58 (0.07-4.81)	0.61	0.84 (0.10-7.00)	0.87
ASA score	≤2	reference		reference	
	>2	0.91 (0.25-3.27)	0.88	0.78 (0.17-3.57)	0.75
Prostate volume	≤25	reference		reference	
	>25	1.28 (0.57-2.89)	0.56	1.50 (0.58-3.90)	0.41
Hernia surgery history	no	reference		reference	
	yes	0.73 (0.16-3.34)	0.68	0.46 (0.06-3.62)	0.46
Pelvic surgery history	no	reference		reference	
	yes	0.63 (0.21-1.90)	0.41	0.63 (0.18-2.23)	0.48
**Tumor**					
T stage	T2	reference		reference	
	≥T3	1.66 (0.77-3.58)	0.19	2.31 (1.00-5.29)	0.05
Gleason	6	reference		reference	
	7	0.98 (0.40-2.39)	0.96	1.24 (0.43-3.64)	0.69
	≥8	2.25 (0.81-6.21)	0.12	2.93 (0.90-9.51)	0.07
NCCN	low	reference		reference	
	intermediate	1.52 (0.46-5.06)	0.49	0.68 (0.19-2.53)	0.57
	high	1.50 (0.45-4.98)	0.51	1.25 (0.37-4.21)	0.72
**Surgical factor**					
Operative time	<120	reference		reference	
	≥120	3.11 (1.39-6.94)	0.0056	4.55 (1.67-12.45)	0.0031
Estimated blood loss	≤50	reference		reference	
	>50	2.43 (1.13-5.24)	0.023	1.97 (0.83-4.68)	0.13
Lymph node dissection	no	reference		reference	
	yes	1.99 (0.60-6.64)	0.26	1.89 (0.50-7.16)	0.35
NVB sparing	no	reference		reference	
	yes	0.65 (0.30-1.38)	0.26	0.62 (0.26-1.47)	0.28
Bladder neck preservation	no	reference		reference	
	yes	0.79 (0.39-1.60)	0.51	0.78 (0.35-1.73)	0.54

Table S2. Univariable analysis of the associations between circulating markers and the primary and secondary outcomes. OR: odds ratio; CI: confidence interval.

	cutoff	Grade II or greater complication		Surgical site infection	
		OR (95% CI)	P	OR (95% CI)	P
NLR (day0-pre)	<11.5	reference		reference	
	≥11.5	5.65 (1.71-18.64)	0.005	3.79 (1.06-13.55)	0.04
PLR (day0-pre)	<50	reference		reference	
	≥50	1.05 (0.42-2.59)	0.92	0.71 (0.23-2.18)	0.55
LMR (day0-pre)	<-1.5	reference		reference	
	≥-1.5	0.49 (0.23-1.02)	0.05	0.27 (0.12-0.61)	0.002
SII (day0-pre)	<2100	reference		reference	
	≥2100	3.88 (1.16-13.00)	0.03	1.38 (0.29-6.68)	0.69
LCR (day0-pre)	<1.8	reference		reference	
	≥1.8	3.02 (1.11-8.18)	0.03	2.54 (0.84-7.63)	0.10
WBC (day0-pre)	<2	reference		reference	
	≥2	2.38 (1.06-5.33)	0.04	4.70 (1.57-14.07)	0.01
RBC (day0-pre)	<0.08	reference		reference	
	≥0.08	0.20 (0.03-1.53)	0.12	0.28 (0.04-2.17)	0.22
HB (day0-pre)	<0	reference		reference	
	≥0	0.75 (0.25-2.30)	0.62	0.46 (0.10-2.06)	0.31
Plt (day0-pre)	<-10	reference		reference	
	≥-10	0.77 (0.36-1.65)	0.50	0.88 (0.38-2.06)	0.77
N (day0-pre)	<2.5	reference		reference	
	≥2.5	2.82 (1.29-6.17)	0.01	4.85 (1.77-13.29)	0.002
L (day0-pre)	<-0.1	reference		reference	
	≥-0.1	1.62 (0.80-3.30)	0.18	1.75 (0.79-3.90)	0.17
M (day0-pre)	<0.07	reference		reference	
	≥0.07	1.76 (0.86-3.59)	0.12	3.60 (1.57-8.26)	0.002
CRP (day0-pre)	<0.5	reference		reference	
	≥0.5	0.48 (0.18-1.32)	0.15	0.53 (0.18-1.62)	0.27

Table S3. Univariable analysis of the associations between circulating markers and the primary and secondary outcomes. OR: odds ratio; CI: confidence interval.

	**cutoff**	**Grade II or greater complication**		**Surgical site infection**	
		**OR (95% CI)**	**P**	**OR (95% CI)**	**P**
NLR (day1-pre)	<5.2	reference		reference	
	≥5.2	2.23 (1.01-4.92)	0.05	2.04 (0.85-4.89)	0.11
PLR (day1-pre)	<100	reference		reference	
	≥100	0.62 (0.22-1.70)	0.35	0.28 (0.06-1.25)	0.10
LMR (day1-pre)	<-3	reference		reference	
	≥-3	0.68 (0.33-1.42)	0.31	0.46 (0.20-1.06)	0.07
SII (day1-pre)	<1500	reference		reference	
	≥1500	0.77 (0.30-1.98)	0.58	0.44 (0.13-1.55)	0.20
LCR (day1-pre)	<-0.75	reference		reference	
	≥-0.75	0.33 (0.11-0.98)	0.05	0.48 (0.16-1.47)	0.20
WBC (day1-pre)	<6.5	reference		reference	
	≥6.5	2.06 (0.83-5.10)	0.12	1.44 (0.50-4.14)	0.50
RBC (day1-pre)	<-1	reference		reference	
	≥-1	0.25 (0.09-0.66)	0.01	0.22 (0.08-0.61)	0.004
HB (day1-pre)	<-30	reference		reference	
	≥-30	0.33 (0.13-0.82)	0.02	0.28 (0.11-0.71)	0.01
Plt (day1-pre)	<-25	reference		reference	
	≥-25	0.63 (0.30-1.35)	0.24	0.51 (0.21-1.22)	0.13
N (day1-pre)	<6	reference		reference	
	≥6	2.09 (0.95-4.60)	0.07	1.91 (0.79-4.58)	0.15
L (day1-pre)	<-0.5	reference		reference	
	≥-0.5	1.05 (0.50-2.20)	0.90	1.08 (0.48-2.47)	0.85
M (day1-pre)	<0.2	reference		reference	
	≥0.2	1.38 (0.67-2.87)	0.38	1.88 (0.83-4.25)	0.13
CRP (day1-pre)	<28	reference		reference	
	≥28	2.69 (1.26-5.74)	0.01	2.17 (0.93-5.06)	0.07

Table S4. Univariable analysis of the associations between circulating markers and the primary and secondary outcomes. OR: odds ratio; CI: confidence interval.

	cutoff	Grade II or greater complication		Surgical site infection	
		**OR (95% CI)**	**P**	**OR (95% CI)**	**P**
NLR (day1-day0)	<5	reference		reference	
	≥5	2.11 (1.00-4.46)	0.05	1.60 (0.70-3.68)	0.27
PLR (day1-day0)	<100	reference		reference	
	≥100	1.01 (0.38-2.67)	0.98	0.57 (0.16-2.02)	0.39
LMR (day1-day0)	<-3.7	reference		reference	
	≥-3.7	1.40 (0.54-3.62)	0.49	3.88 (0.88-17.10)	0.07
SII (day1-day0)	<500	reference		reference	
	≥500	0.87 (0.42-1.80)	0.70	0.61 (0.27-1.38)	0.23
LCR (day1-day0)	<-2.5	reference		reference	
	≥-2.5	0.74 (0.34-1.60)	0.45	0.73 (0.31-1.73)	0.47
WBC (day1-day0)	<-0.5	reference		reference	
	≥-0.5	0.50 (0.23-1.06)	0.07	0.35 (0.15-0.79)	0.01
RBC (day1-day0)	<-0.2	reference		reference	
	≥-0.2	0.95 (0.45-1.97)	0.88	0.86 (0.38-1.95)	0.71
HB (day1-day0)	<-12	reference		reference	
	≥-12	0.52 (0.22-1.23)	0.14	0.53 (0.20-1.37)	0.19
Plt (day1-day0)	<-25	reference		reference	
	≥-25	1.82 (0.52-6.40)	0.35	0.88 (0.28-2.78)	0.83
N (day1-day0)	<-1.2	reference		reference	
	≥-1.2	0.40 (0.17-0.95)	0.04	0.40 (0.16-1.02)	0.05
L (day1-day0)	<-0.5	reference		reference	
	≥-0.5	0.98 (0.47-2.05)	0.96	0.91 (0.40-2.07)	0.83
M (day1-day0)	<0.2	reference		reference	
	≥0.2	0.98 (0.46-2.09)	0.96	0.98 (0.42-2.26)	0.96
CRP (day1-day0)	<29	reference		reference	
	≥29	2.97 (1.38-6.38)	0.01	2.37 (1.01-5.55)	0.05
